# A Heat-Shock Transcription Factor in *Panax ginseng*, *PgHSFA2*, Confers Heat and Salt Resistance in Transgenic Tobacco

**DOI:** 10.3390/ijms26083836

**Published:** 2025-04-18

**Authors:** Sung Won Jeon, Yi Rae Kim, Jung Yeon Han, Ukhan Jeong, Eun Ju Cheong, Yong Eui Choi

**Affiliations:** Division of Forest Sciences, College of Forest and Environmental Sciences, Kangwon National University, Chuncheon 200-701, Republic of Korea; jeonsw1211@naver.com (S.W.J.); yiraekim@kangwon.ac.kr (Y.R.K.); jyhan@kangwon.ac.kr (J.Y.H.); uki7291@naver.com (U.J.); ejcheong@kangwon.ac.kr (E.J.C.)

**Keywords:** *PgHSFA2*, *Panax ginseng*, heat-shock transcription factor, abiotic stress tolerance, transgenic tobacco

## Abstract

*Panax ginseng* plants are susceptible to high temperatures and intense sunlight, necessitating cultivation under artificially shaded structures. Identifying the genes associated with heat resistance is critical for advancing molecular breeding strategies to develop heat-tolerant ginseng varieties. Heat-shock transcription factors (HSFs) are widely recognized as key regulators of plant responses to abiotic stresses, primarily by controlling heat-shock proteins (HSPs). To identify HSF genes in *P. ginseng*, transcriptome analysis was conducted on ginseng plants subjected to heat-shock treatment (1 h at 40 °C). Among the 26 HSF unigenes annotated from the ginseng transcriptome, a unigene related to the HSFA2 family exhibited the highest transcriptional activity following heat-shock treatment. The expression of *PgHSFA2*, a gene identified from this unigene, was analyzed under temperature and salt-stress conditions in ginseng plants using qPCR. The results showed that *PgHSFA2* was highly responsive to various abiotic stresses, including heat, cold, salt, and intense sunlight. To assess the functional role of *PgHSFA2*, transgenic tobacco plants overexpressing this gene were analyzed. The overexpression of *PgHSFA2* led to an elevated expression of heat-shock proteins (HSPs) in tobacco, resulting in enhanced resistance to high temperature and salt stress. Transgenic tobacco plants exhibited significantly less reduction in chlorophyll fluorescence compared to nontransgenic controls when exposed to salt stress (200 and 400 mM NaCl) and high-temperature stress (42 °C), indicating improved stress tolerance. In conclusion, *PgHSFA2* is a crucial HSF that regulates the transcriptional control of HSPs in ginseng plants. The constitutive expression of *PgHSFA2* in transgenic ginseng could potentially confer improved tolerance to high temperatures, making it a valuable target for molecular breeding.

## 1. Introduction

Heat-shock transcription factors (HSFs) play a critical role in the cellular response to various stressors, particularly heat stress. HSFs regulate the expression of heat-shock proteins (HSPs), a group of stress-responsive genes that help cells mitigate adverse conditions [1]. Also referred to as stress proteins, HSPs are vital for protecting and maintaining the integrity of cellular proteins, ensuring cell survival under environmental stresses such as heat [2]. HSFs bind to cis-acting elements in the promoter regions of *HSP* genes, directly regulating their expression. In many plant species, the overexpression of *HSFs* has been shown to enhance resistance to abiotic stresses [3,4,5,6,7,8,9].

In the model plant *A. thaliana*, 21 distinct HSFs have been identified and categorized into three major classes: A, B, and C [2,10]. Among these, HsfA1 is the primary positive regulator of heat-shock-responsive gene expression [6]. However, its overexpression shows limited defensive effects compared to HsfA2 or HsfA3 [11]. HsfA2, in particular, forms heteromeric complexes with HsfA1 proteins to regulate HSP expression [12]. Its mRNA levels significantly increase following heat-stress treatment [3]. As a key TF, HsfA2 regulates defensive genes under various environmental stresses, and its overexpression in *Arabidopsis* has been widely reported to confer enhanced tolerance to abiotic stresses [3,4,5,13].

*Panax ginseng* is considered one of the most valuable medicinal plants in the Orient. However, its cultivation poses significant challenges due to its inherent susceptibility to biotic and abiotic stress conditions. Ginseng plants are particularly vulnerable to high temperatures and intense sunlight, necessitating sunshade facilities to mitigate heat- and light-stress damage. Thus, heat stress remains a significant issue for ginseng cultivation, especially as global warming exacerbates temperature extremes. High temperatures are a key environmental factor reducing crop productivity [14,15]. Given the central role of HSFs in plant thermotolerance, understanding the molecular mechanisms of HSF genes in *P. ginseng* is crucial. However, no studies have functionally analyzed HSF genes in ginseng plants. In contrast, WRKY TFs, which also respond to temperature stress, have been identified in ginseng [16]. Genome-wide analyses of WRKY TFs and their responses to abiotic stresses, including heat, have been reported [17].

Transcriptome analysis is a powerful approach for identifying and selecting target genes. In ginseng, transcriptome studies have been conducted on saponin biosynthesis and stress responses [16,18]. Previous research investigated transcriptomic changes in ginseng exposed to moderately high temperatures (30 °C) for one to three weeks [16]. In contrast, heat-shock treatments in *Arabidopsis* typically involve short-term exposure to higher temperatures (37 °C or above) [19]. No transcriptome analyses of ginseng have been conducted under short-term heat-shock conditions.

In this study, we analyzed the expression changes in both HSF and HSP genes in ginseng plants following heat-shock treatment. We identified ***PgHSFA2***, a key HSF gene, as the central regulator of HSPs in ginseng and evaluated its expression under various abiotic stress conditions. Furthermore, transgenic tobacco plants overexpressing ***PgHSFA2*** exhibited strong tolerance to heat and salt stress, highlighting its potential for improving stress resistance in ginseng.

## 2. Results

### 2.1. Transcriptome Analysis of Ginseng Plants After Heat-Shock Treatment

**Figure 1:**
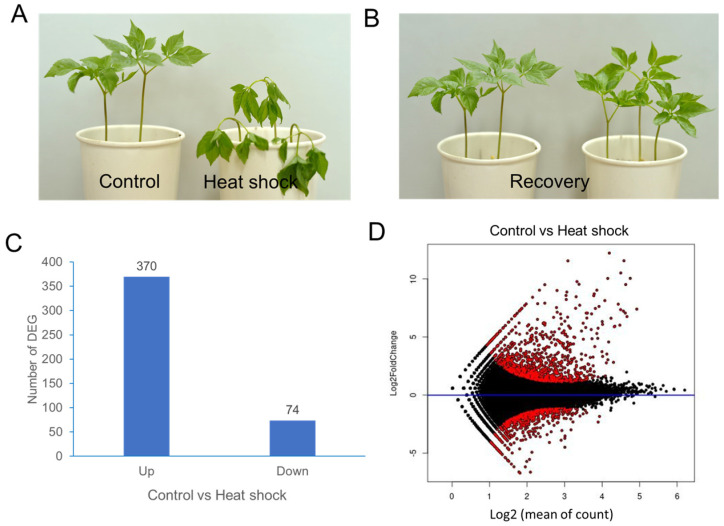
A transcriptome analysis of *P. ginseng* leaves after heat-shock treatment. (**A**) *P. ginseng* plants immediately after heat-shock treatment at 40 °C for 1 h. (**B**) *P. ginseng* plants 24 h after recovery at 25 °C. (**C**) The number of up/downregulated DEGs. (**D**) MA plot showing differential expression analysis of RNA-seq samples. The y-axis represents the log2-fold change, and the x-axis represents the normalized mean expression. Red points indicate the most significantly differentially expressed genes (DEGs).

Two-year-old ginseng plants were subjected to heat-shock treatment at 40 °C for 1 h or maintained under control conditions without heat shock (Figure 1A,B). Transcriptome analysis of ginseng leaf samples was conducted by sequencing (details can be found in the Supplementary Materials). De novo assembly produced 40,088 unigenes from 174,999 contigs. Annotation of the 40,088 unigenes was performed using the NCBI nonredundant protein sequence (NR) database and the InterPro database (ebi.ac.uk/InterPro/search/sequence). Transcript abundance for each unigene was normalized using DESeq2, and differential gene expression (DEG) analysis was conducted.

The mean-average (MA) plot analysis illustrates the average log-expression changes in ginseng leaves with and without heat-shock treatment (Figure 1C). DEG analysis identified 370 upregulated and 74 downregulated unigenes, using criteria of log2-fold change ≥ +2 and ≤−2, respectively (Figure 1D).

**Figure 2:**
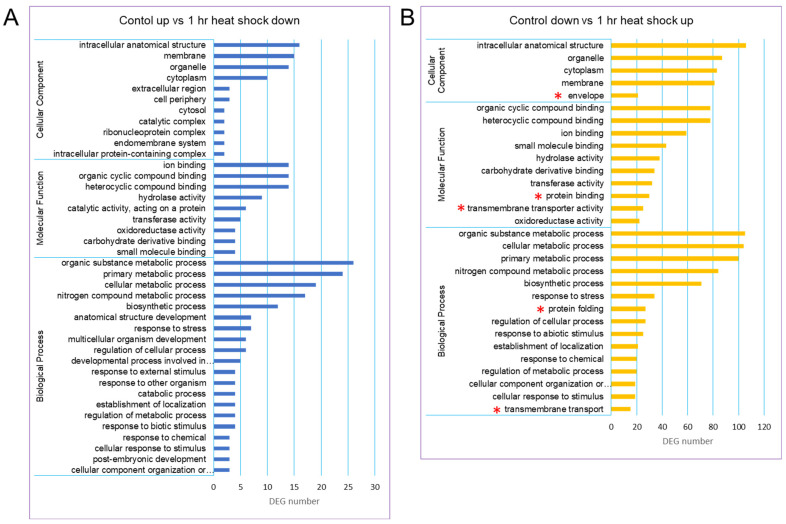
GO classification of the assembled unigenes in the ginseng transcriptome. Three main GO categories were shown in BP, CC, MF. (**A**) GO classification of control up vs. 1 h heat-shock down. (**B**) GO classification of control down vs. 1 h heat-shock up. The GO terms were shown at the vertical axis, the gene number were given at the horizontal axis. Asterisks in red indicate the new representation of subcategories in the heat-shock-treated group.

Based on gene ontology (GO) terms, a total of 40,088 unigenes could be classified into 3 GO categories such as biological process (BP), molecular function (MF), and cellular component (CC). There were more unigenes classified into BP than the other two categories. A GO distribution analysis of gene groups revealed that there was a new representation of “envelope” in the category CC, “protein binding and transmembrane transporter activity” in the category MF, and “protein folding and transmembrane transport” in the category BP (Figure 2).

The top 15 unigenes with the highest log2-fold changes (≥8.5) after heat-shock treatment are listed in Table S2. Among these, the unigene annotated as a 22.0 kDa HSP-like protein displayed the greatest log2-fold change (12.2). Of the 15 highly expressed unigenes, 6 were HSP genes, including two *HSP22 kDa*, *HSP70 kDa*, two *HSP17.3 kDa*, and *HSP32 kDa*. Other unigenes with high log2-fold changes included *pyridoxal 5’-phosphate synthase-like*, *protein BOBBER 1*, *putative nuclease HARBI1*, and *chloroplastic pentatricopeptide repeat-containing protein*, all showing log2-fold changes above 8.5.

### 2.2. Transcriptional Profiling of HSF Unigenes in Response to Heat-Shock Treatment

**Figure 3:**
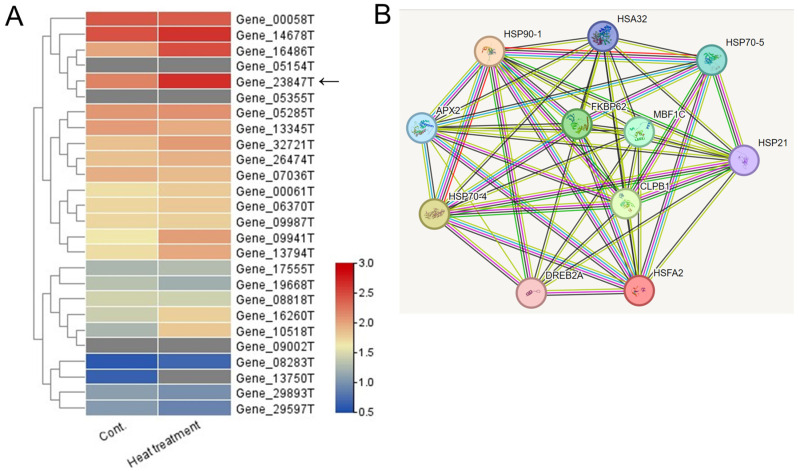
Heatmap and predicted protein–protein interaction network of 26 annotated HSF unigenes in ginseng leaves. (**A**) A heatmap analysis illustrating the expression levels of (DEGs among 26 annotated HSF unigenes under control and heat-shock treatment conditions. Expression levels are represented with a color gradient, where red denotes high expression and blue denotes low expression. An arrow indicates a unigene showing the greatest increase in expression following heat-shock treatment. (**B**) Predicted protein–protein interaction network involving the predicted ORF of a TF unigene (*Gene_23847T*) and associated HSPs. Homologous proteins from *Arabidopsis thaliana* were used as references. Edges represent predicted protein–protein associations, with the analysis conducted using STRING v11.0 and a minimum interaction score set to “medium confidence” (0.3).

Among the 40,088 unigenes identified, 26 were annotated as HSF unigenes (Table S3). Heatmap visualization based on FPKM values revealed that the unigene with the highest log2-fold change after heat-shock treatment was *Gene_23847T* (Figure 3A). This HSF gene was found to be closely related to the *Arabidopsis HsfA2* gene, which is known as a key regulator of heat-stress responses in *Arabidopsis* [3,4]. Thus, the *Gene_23847T* unigene is named the *PgHSFA2* gene.

We performed a correlation analysis among the PgHSFA2 protein and the orthologs of Arabidopsis proteins based on the protein interaction network obtained from STRING database (Figure 3B). The predicted protein of PgHSFA2 is interacting with several HSPs (HSP21, HSP32, HSP70-4, HSP70-5, and HSP90-1), and two other proteins involved in protein folding, FKBP62 (FK506-binding protein 62), and CLPB1 (Caseinolytic Protease B1). PgHSFA2 also interacts with TFs, DREB2A (dehydration-responsive element-binding protein 2A), and MBF1C (multiprotein bridging factor 1C), critical in abiotic stress responses, particularly heat and drought stress.

### 2.3. Transcriptional Profiling of HSP Unigenes in Response to Heat-Shock Treatment

HSPs are a family of proteins produced in response to heat stress. A total of 106 unigenes were annotated as HSPs among the 40,088 unigenes identified (Table S4). Of these, 25 unigenes displayed an upregulated log2-fold change greater than 2.0. The top five most highly expressed unigenes following heat-shock treatment were *Gene_22767T* (22.0 kDa HSP-like), *Gene_02936T* (70 kDa HSP-like), *Gene_33681T* (22 kDa HSP-like), *Gene_30195T* (17.3 kDa HSP-like), and *Gene_31940T* (32 kDa HSP-like). These five unigenes exhibited log2-fold changes exceeding 9.0 after heat-shock treatment.

### 2.4. Isolation of the HSFA2 in P. ginseng

**Figure 4:**
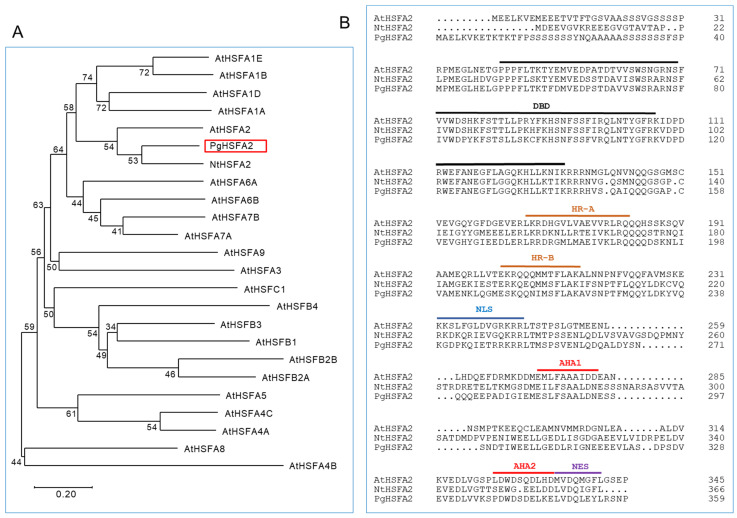
A phylogenetic and ClustalW analysis of *P. ginseng PgHSFA2* and other plant HSFA proteins. (**A**) A phylogenetic tree of *PgHSFA2* and other plant HSFA proteins. *PgHSFA2* is highlighted with a red box. (**B**) ClustalW alignment of the amino-acid sequence of *PgHSFA2* with *AtHSFA2* (from *A. thaliana*) and *NtHSFA2* (from *N. tabacum*). The conserved domains are labeled DBD (DNA-binding domain), HR-A and HR-B (hydrophobic heptad repeats), NLS (nuclear localization signal), AHA (aromatic, large hydrophobic, and acidic amino-acid residues), and NES (nuclear export signal). NCBI protein accession numbers: *P. ginseng PgHSFA2*, OQ134777; *A. thaliana AtHSFA2*, AEC07800; *N. tabacum NtHSFA2*, XP_004985605.1.

The open reading frame (ORF) of *Gene_23847T* was obtained through resequencing after PCR amplification and named *PgHSFA2* (GenBank accession number OQ134777). A phylogenetic analysis of the deduced amino-acid sequences showed that *PgHSFA2* clustered with *AtHSFA2* (from *A. thaliana*) and *NtHSFA2* (from *Nicotiana tabacum*) (Figure 4A). The deduced amino-acid sequence of *PgHSFA2* shared characteristics with *NtHSFA2* and *AtHSFA2* of 62.43% and 53.4%, respectively.

All HSFs are characterized by a helix–loop–helix DNA-binding domain (DBD), which binds to heat-shock elements in the promoter regions of HSP genes. The oligomerization domain, comprising the heptad repeat A (HR-A) and heptad repeat B (HR-B), enables the formation of various oligomers with other HSFs, which is critical for individual defensive strategies under abiotic stress conditions [20]. In *Arabidopsis*, most class A HSFs also contain activation domains (AHA motifs) in their C-terminal regions, which are essential for transcriptional activation, while class B and C HSFs lack these motifs [21,22].

The deduced amino-acid sequence of *PgHSFA2* contains conserved domains typical of HSFs, including the DBD, HR-A/-B, nuclear localization signal (NLS), nuclear export signal (NES), and two AHA motifs (Figure 4B). These findings confirm that *PgHSFA2* belongs to the A2 subclass of HSF genes.

### 2.5. Expression of PgHSFA2 in the Leaves of Ginseng Under Various Stress Treatments

**Figure 5:**
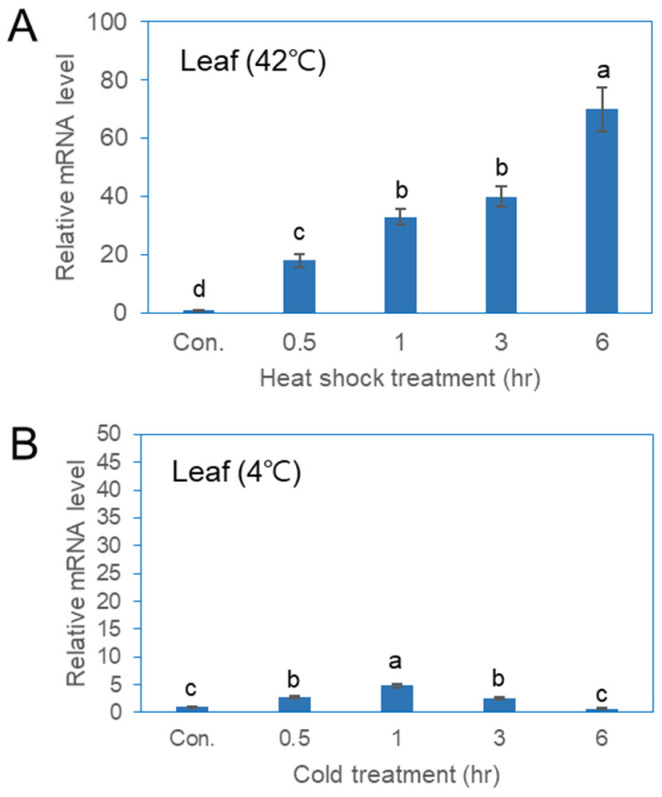
qRT-PCR analysis of *PgHSFA2* expression in *P. ginseng* under various environmental stresses. (**A**) *PgHSFA2* expression in leaves after treatment at 42 °C for 0.5, 1, 3, and 6 h. (**B**) *PgHSFA2* expression in leaves after treatment at 4 °C for 0.5, 1, 3, and 6 h. The ginseng β-actin gene was used for normalization. The data presented are the mean ± SE of the transcripts obtained from at least three independent experiments. Bars marked with different letters denote statistically significant differences (*p* < 0.05, one-way ANOVA).

qPCR analysis was conducted to evaluate the expression of the *PgHSFA2* gene in ginseng plants under various abiotic stress conditions (Figure 5). The expression of *PgHSFA2* in leaves increased rapidly between 0.5 and 6 h of heat-shock treatment (Figure 5A). While *PgHSFA2* was also responsive to cold stress, the expression level was lower compared to that of heat-shock treatment (Figure 5B). Under cold conditions (4 °C), *PgHSFA2* expression peaked after 1 h and subsequently decreased (Figure 5B).

### 2.6. Construction of Transgenic Tobacco Plants Overexpressing the PgHSFA2 Gene

**Figure 6:**
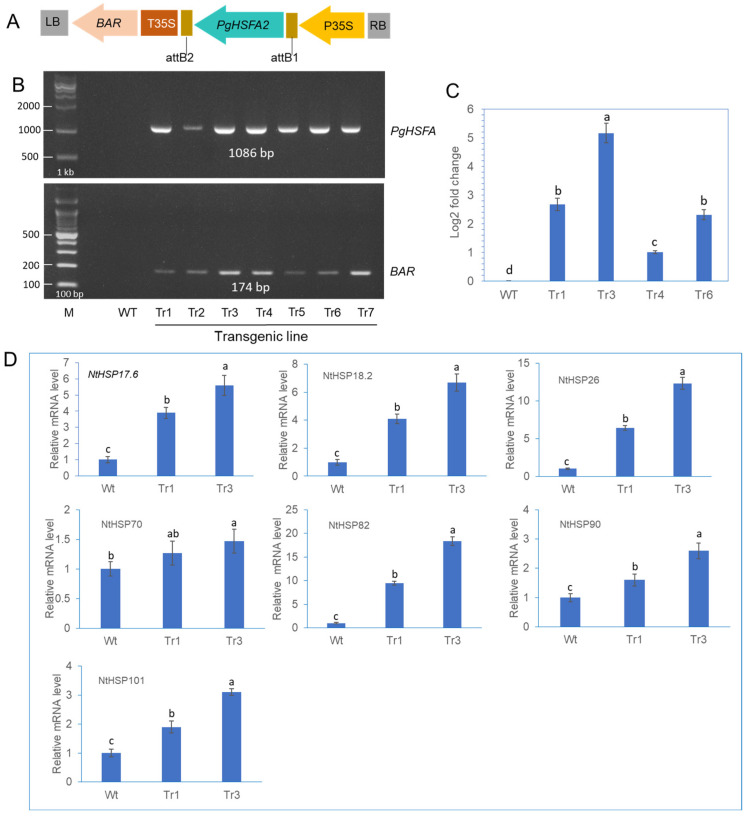
The construction of transgenic tobacco overexpressing *PgHSFA2* and expression analysis of tobacco HSPs in transgenic lines. (**A**) A schematic representation of the vector expressing *PgHSFA2* under the control of the CaMV35S promoter. (**B**) Genomic PCR detection of the *PgHSFA2* gene (upper panel) and the *BAR* gene (lower panel) in transgenic tobacco lines. (**C**) qRT-PCR analysis of *PgHSFA2* expression in four transgenic lines of tobacco. Tobacco actin gene RT-PCR products are shown as a loading control. Data are presented as average relative quantities ± standard errors (SEs). (**D**) qRT-PCR analysis of tobacco HSP genes (*NtHSP17.6*, *NtHSP18.2*, *NtHSP26*, *NtHSP70*, *NtHSP82*, *NtHSP90*, and *NtHSP101*) in transgenic tobacco under ambient temperature (25 °C). Data are presented as average relative quantities ± standard errors (SEs). Bars marked with different letters denote statistically significant differences (*p* < 0.05, one-way ANOVA).

Transgenic tobacco plants were generated by infecting leaf segments with *Agrobacterium tumefaciens* carrying the *PgHSFA2* and *BAR* (BASTA resistance) genes (Figure 6A). To identify putative transgenic plants overexpressing *PgHSFA2*, genomic PCR was performed (Figure 6B). Among the seven tested lines, transgenic lines Tr1, Tr3, Tr4, and Tr6 were selected for further analysis. The qRT-PCR analysis revealed that *PgHSFA2* expression was highest in Tr3 among the four transgenic lines (Figure 6C).

### 2.7. Expression of Tobacco HSP Genes in Transgenic Tobacco Overexpressing PgHSFA2

To investigate the regulatory role of *PgHSFA2* overexpression in transgenic tobacco on the expression of intrinsic tobacco HSP genes, the expression levels of seven *NtHSP* genes (*NtHSP17.6*, *NtHSP18.2*, *NtHSP26*, *NtHSP70*, *NtHSP82*, *NtHSP90*, and *NtHSP101*) were analyzed by qPCR. Under ambient growth conditions, six *NtHSPs* were significantly upregulated in transgenic lines overexpressing *PgHSFA2* compared to wild-type plants, except *NtHSP70* (Figure 6D). Among these, *NtHSP82* exhibited the highest expression levels (Figure 6D).

### 2.8. Viability Assays Under Heat Stress and High Salinity Stress

**Figure 7:**
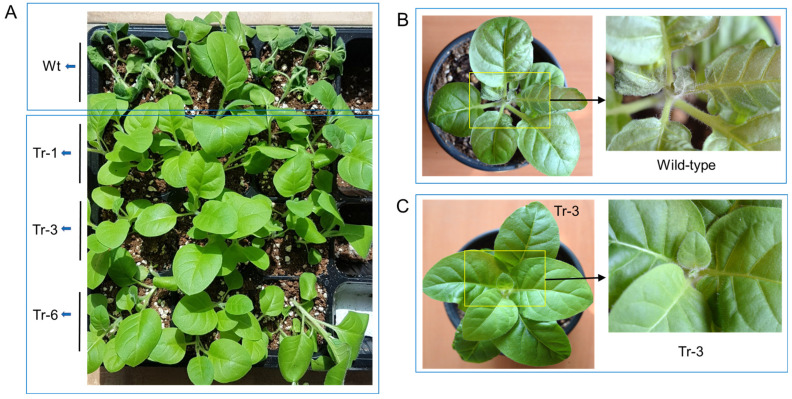
Heat resistance in transgenic tobacco overexpressing *PgHSFA2*. (**A**–**C**) Recovery of wild-type and transgenic tobacco plants after heat-shock treatment. (**A**) Heat-shock treatment at 45 °C for 3 h in 2-month-old wild-type and transgenic tobacco plants. Wilted wild-type plants are shown at the top, while healthy transgenic lines (Tr-1, Tr-3, and Tr-6) are shown at the bottom after 3 days of recovery. (**B**,**C**) Heat-shock treatment at 50 °C for 1 h in 3-month-old wild-type (**B**) and transgenic (Tr-3) tobacco plants (**C**) after 3 days of recovery. Enlarged views of the rectangular regions are shown on the right.

One-month-old transgenic tobacco plants (Tr-1, Tr-3, and Tr-6) and wild-type plants were subjected to high-temperature stress (45 °C for 3 h). After a 3-day recovery period, wild-type plants exhibited severe leaf wilting. In contrast, the leaves of transgenic tobacco plants had recovered to a healthy state (Figure 7A). Similarly, when two-month-old wild-type and transgenic (Tr-3) tobacco plants were treated at 50 °C for 1 h, wild-type plants showed significant leaf damage, particularly at the shoot tips, after 3 days of recovery (Figure 7B). In contrast, Tr-3 plants maintained a healthy appearance in both leaves and shoot tips (Figure 7C).

**Figure 8:**
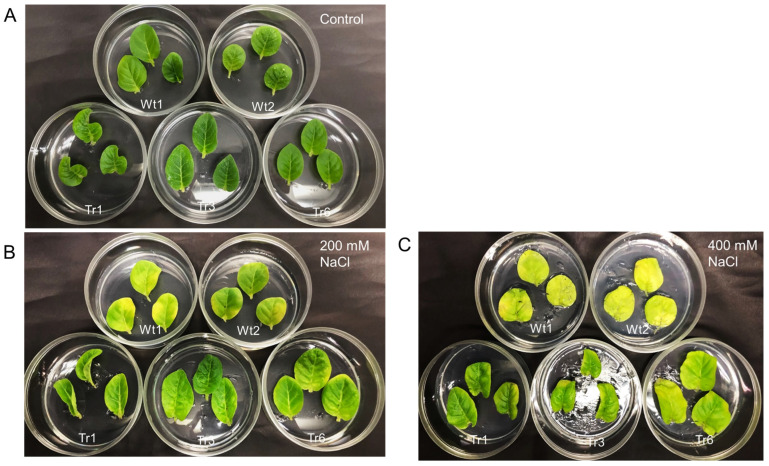
Salt resistance in transgenic tobacco overexpressing *PgHSFA2* under different NaCl concentrations. Two wild-type plants are shown at the top, while three transgenic lines (Tr-1, Tr-3, and Tr-6) are shown at the bottom. (**A**) Leaves after 9 days in medium without NaCl. (**B**) Leaves after 9 days in medium containing 200 mM NaCl. (**C**) Leaves after 9 days in medium containing 400 mM NaCl.

To examine the response to salt stress in transgenic tobacco, detached leaves from transgenic lines and wild-type plants were placed on half-strength MS medium with varying salinity levels (0, 200, or 400 mM NaCl). After 9 days, no noticeable differences in leaf color were observed between transgenic and wild-type plants in the absence of NaCl treatment (Figure 8A). However, under 200 and 400 mM NaCl treatments, leaves from wild-type plants were more etiolated than those of the transgenic lines (Figure 8B,C).

### 2.9. Change in Chlorophyll Contents and Maximum Quantum Yield of PSII in Transgenic Tobacco Overexpressing PgHSFA2

**Figure 9:**
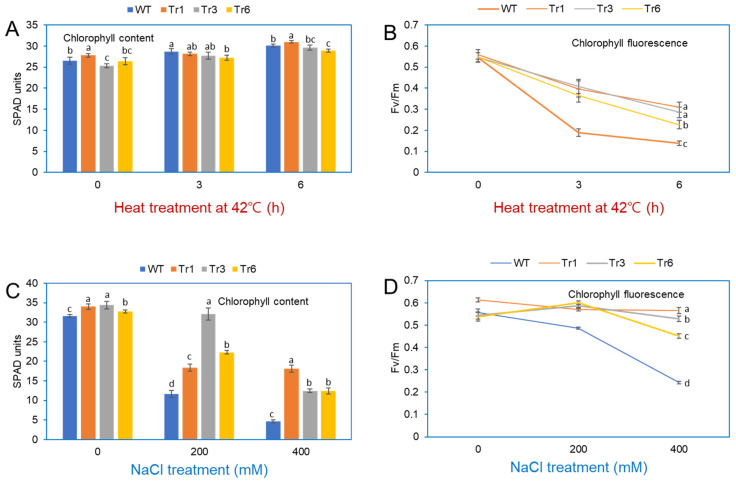
Chlorophyll content and fluorescence in leaves of wild-type and transgenic tobacco overexpressing *PgHSFA2* after heat and salt treatment. (**A**) Chlorophyll content in leaves after heat treatment at 42 °C for 0, 3, and 6 h. (**B**) Fv/Fm measurements (maximum quantum efficiency of Photosystem II) after heat treatment at 42 °C for 0, 3, and 6 h. Data are presented as average relative quantities ± standard errors (SEs). (**C**) Chlorophyll content in leaves after salt treatment with 0, 200, and 400 mM NaCl. (**D**) Fv/Fm measurements after salt treatment with 0, 200, and 400 mM NaCl. Data are presented as average relative quantities ± standard errors (SEs). Bars marked with different letters denote statistically significant differences (*p* < 0.05, one-way ANOVA).

Stress exposure in plants negatively impacts PSII and PSI, leading to a decrease in photosynthetic efficiency [23]. Under heat treatment (42 °C for 3 and 6 h), the chlorophyll content in the leaves of both transgenic and wild-type tobacco plants remained unchanged (Figure 9A). Fv/Fm, a standard parameter for assessing plant stress and the health of Photosystem II [24], revealed differences in photosynthetic efficiency between transgenic and wild-type plants after heat stress. At 25 °C, Fv/Fm values were similar in transgenic and nontransgenic tobacco. However, after heat-shock treatment at 42 °C, Fv/Fm values in wild-type plants decreased rapidly after 3 and 6 h, whereas transgenic plants showed a more gradual decline over the same period (Figure 9B).

The chlorophyll content in wild-type and transgenic tobacco plants overexpressing *PgHSFA2* was also assessed after NaCl treatment. In the absence of NaCl, chlorophyll levels were comparable between transgenic and wild-type plants. However, under 200 or 400 mM NaCl treatments, wild-type plants exhibited a significant reduction in chlorophyll content compared to transgenic lines, with the most pronounced differences observed at 400 mM NaCl (Figure 9C). The maximum quantum yield of PSII (Fv/Fm) remained relatively stable (0.45–0.52) in transgenic plants but decreased sharply in wild-type plants under salt stress (Figure 9D).

## 3. Discussion

HSFs and HSPs are crucial components of the heat-stress response in plants. Their primary function is to protect cells from the damaging effects of high temperatures by maintaining protein homeostasis. HSFs are key regulators of plant responses to heat stress by modulating HSP expression [1].

Transcriptome sequencing was conducted to analyze the transcriptional activity of HSF and HSP genes in *P. ginseng* plants exposed to heat shock. Heat-shock treatment significantly altered the expression levels (log2-fold change) of HSF and HSP unigenes. Among the 26 annotated HSF unigenes, *Gene_23847T*, corresponding to *PgHSFA2*, exhibited the highest expression level after heat shock. Arabidopsis contains 21 HSF genes, which are categorized into three classes based on their structural features and regulatory roles: HSFA, HSFB, and HSFC. HSFAs primarily function as transcriptional activators of heat-responsive genes. HSFA1 functions as the primary heat-stress regulator, rapidly activating early heat-responsive genes and initiating the plant’s basal thermotolerance. In contrast, HSFA2 is induced by HSFA1 and plays a key role in sustaining the heat response during prolonged or repeated stress, contributing to acquired thermotolerance and stress memory [3,4,5,13].

HSPs are a family of proteins and transcriptionally regulated by HSFs, which act as key transcription factors during abiotic stress, especially heat stress. A total of 106 unigenes were annotated as HSPs among the 40,088 unigenes identified (Table S4). Of these, 25 unigenes displayed an upregulated log2-fold change greater than 2.0. The top five most highly expressed unigenes following heat-shock treatment were *Gene_22767T* (22.0 kDa HSP-like), *Gene_02936T* (70 kDa HSP-like), *Gene_33681T* (22 kDa HSP-like), *Gene_30195T* (17.3 kDa HSP-like), and *Gene_31940T* (32 kDa HSP-like). These five unigenes exhibited log2-fold changes exceeding 9.0 after heat-shock treatment. *Gene_22767T*, belonging to 22.0 kDa HSP, displayed the highest expression value among the various HSP genes. The 22.0 kDa HSP is classified as a member of the small HSP family, and is known to play a crucial role in thermotolerance, cellular homeostasis, and the mitigation of oxidative stress [25,26], indicating its importance in ginseng leaves under heat stress.

In this study, *PgHSFA2* was one of the most responsive HSFs under heat-shock stress in *P. ginseng.* The deduced amino-acid sequence of *PgHSFA2* shared 53.4% identity with *AtHsfA2*. Notably, *PgHSFA2* contained two AHA motifs in its C-terminal region, a characteristic feature of class A HSFs, which are absent in class B and C HSFs [21,22], confirming its classification as a class A HSF.

The transcriptional activity of *PgHSFA2* in ginseng plants was further analyzed under various abiotic stress conditions. qPCR analysis revealed that *PgHSFA2* was faintly expressed under normal conditions but strongly induced by heat-shock treatment at 42 °C. However, cold treatment at 4 °C resulted in a slight increase in the expression of *PgHSFA2* up to 1 h, followed by a decrease over time. Similarly, *AtHsfA2* expression in *Arabidopsis* is negligible under normal conditions but significantly upregulated by heat shock [4,5,27].

HSFs bind to heat-shock elements in the promoter regions of HSP genes, initiating their transcription. Under heat-shock stress, HSPs play a crucial protective role by maintaining protein homeostasis and promoting cell survival. The newly synthesized HSPs then function as molecular chaperones, refolding denatured proteins and protecting cells from heat-induced damage [27,28,29]. Proteomic analysis has demonstrated increased expression of various HSPs in ginseng under heat stress [30], and transcriptomic studies indicate that excess light stress also elevates HSP gene expression in *P. ginseng* [31].

To assess the functional role of *PgHSFA2*, we overexpressed it in tobacco plants and analyzed changes in HSP gene expression and stress tolerance in transgenic tobacco. Transgenic tobacco plants overexpressing *PgHSFA2* exhibited strong resistance to high-temperature stress (45 °C for 3 h or 50 °C for 2 h), suggesting that *PgHSFA2* overexpression enhances heat tolerance in plants. qPCR analysis showed that all seven tobacco HSP genes (*NtHSP17.6*, *NtHSP18.2*, *NtHSP70*, *NtHSP82*, *NtHSP90*, and *NtHSP101*) were upregulated in transgenic lines compared to wild-type plants at normal growth temperatures (25 °C). In *Arabidopsis* overexpressing *HsfA2*, many HSPs were highly expressed compared to wild-type plants [27]. Interestingly, in tobacco overexpressing *PgHSFA2, NtHSP82* showed the highest expression among other *NtHSPs.* HSP82, a specific isoform of HSP90, may be a highly inducible member of the HSP90 family in tobacco. The high expression of *NtHSP82* in transgenic tobacco overexpressing *PgHSFA2* may indicate that the HSE motifs in the HSP82 promoter might be more responsive to HSFA2 binding than those in other HSP genes. Interestingly, among the analyzed seven *NtHSP* genes (*NtHSP17.6*, *NtHSP18.2*, *NtHSP26*, *NtHSP70*, *NtHSP82*, *NtHSP90*, and *NtHSP101*), the transcriptional activity of *NtHSP70* showed a less significant increase in transgenic tobacco compared to others. HSFA1 primarily regulates HSP70 during the initial heat-shock response in Arabidopsis [4]. HSFA2 acts as a secondary regulator, sustaining the expression of certain HSPs during prolonged stress. Therefore, the overexpression of *HSFA2* may not significantly enhance HSP70 levels beyond what is already induced by *HSFA1* [4].

Heat stress impacts chloroplast function, reducing photosynthesis efficiency. Chlorophyll, a critical photosynthetic pigment, largely determines a plant’s photosynthetic capacity. Fv/Fm, a measure of the maximum quantum efficiency of Photosystem II, is an indicator of photosynthetic health, with lower values indicating stress or photoinhibition [28]. Our results showed that transgenic tobacco overexpressing *PgHSFA2* maintained relatively stable chlorophyll content and Fv/Fm values under heat and salinity stress compared to wild-type plants. This indicates that *PgHSFA2* alleviates damage to the photosynthetic apparatus under stress conditions.

Several studies have further elucidated the role of HSFA2 overexpression in enhancing stress tolerance in transgenic plants. The wheat gene *TaHsfA2-13*, when overexpressed in Arabidopsis, conferred improved tolerance to heat stress, oxidative stress (H_2_O_2_), salicylic acid (SA), and mannitol [32]. The overexpression of HSFA2 in Arabidopsis seedlings led to increased expression of several known targets of this transcription factor, resulting in markedly improved tolerance to anoxia and submergence [33]. The overexpression of HSFA2 improves thermomemory more profoundly in specific genetic backgrounds, indicating its role in activating memory-supporting and memory-resetting genes [34]. A regulatory loop involving HSFA2 and the H3K27me3 demethylase REF6 orchestrates transgenerational thermomemory in Arabidopsis. The overexpression of HSFA2-MYC in transgenic plants led to early flowering and elevated expression of SGIP1, a gene involved in stress responses [35]. These studies underscore the multifaceted role of HSFA2 in plant stress responses, including its involvement in anoxia tolerance, thermomemory regulation, transgenerational stress adaptation, and the fine-tuning of heat-stress responses through alternative splicing.

In conclusion, the overexpression of *PgHSFA2* in transgenic tobacco enhances tolerance to heat and salt stresses by regulating HSP expression and protecting the photosynthetic apparatus. These findings suggest that transgenic ginseng overexpressing *PgHSFA2* could lead to a genetically improved variety with enhanced stress tolerance, offering potential for agricultural applications under challenging environmental conditions.

## 4. Materials and Methods

### 4.1. Plant Materials and Growth Conditions

Two-year-old *P. ginseng* plants were sourced from Health of Today, a smart farm company based in Chuncheon, Gangwon, Republic of Korea. Each plant was transplanted into a pot measuring 90 mm in diameter and 165 mm in height. The plants were maintained in a growth chamber under controlled conditions, with a 16/8 h day/night photoperiod at a constant temperature of 25 °C.

Sterilized seeds of *Nicotiana tabacum* (cv. Xanthi) were sown on Petri dishes containing half-strength MS medium [36], supplemented with 2% sucrose and 0.3% Gelrite. The germinated seedlings were transferred to Magenta boxes filled with the same medium and cultured in a growth chamber set to 24 °C with a 16/8 h day/night photoperiod.

### 4.2. RNA Isolation and cDNA Library Construction

For the heat-shock treatment, potted two-year-old *P. ginseng* plants were placed in an incubator at 40 °C for 1 h. Leaves from control and heat-shock-treated plants were immediately frozen in liquid nitrogen. Total RNA was extracted from *P. ginseng* leaves, both with and without heat-shock treatment, using the RNeasy Plant Mini Kit (Qiagen, Hilden, Germany), following the manufacturer’s protocol. For the heat-shock treatment, leaves were collected from three independent plants after 1 h of exposure to 40 °C (total of three plants). As a control, leaves at the same developmental stage were sampled from three independent plants maintained under standard growth conditions at 24 °C in a growth chamber (total of three plants). After assessing the yield and quality of the extracted RNA, the samples were pooled for RNA sequencing (RNA-seq). Two RNA libraries (control and heat-shock-treated at 40 °C for 1 h) were prepared using the Illumina TruSeq RNA Sample Preparation Kit (Illumina, CA, USA). Library sequencing was performed on the Illumina HiSeq X Ten System (Illumina, San Diego, CA, USA).

### 4.3. De Novo Assembly

De novo assembly of the trimmed sequence reads was performed using the assembler tool of Trinity V2.13.2 (https://github.com/trinityrnaseq/trinityrnaseq/wiki). The Trinity assemblies were then optimized for filtering using CD-HIT-EST V4.8.1 (http://weizhong-lab.ucsd.edu/cd-hit/), Bwa V0.7.17 (https://github.com/lh3/bwa), and SAMtools V1.13 (http://www.htslib.org/). Coding regions were found using TransDecoder V5.5.0 (https://github.com/TransDecoder/TransDecoder/wiki) after matching ORF sequences in the UniProt and Pfam databases. Raw sequencing data were deposited in the National Center for Biotechnology Information (NCBI) under the accession number PRJNA1012566. A total of 44,137 unigenes were annotated using the nr (nonredundant) protein database of NCBI BLAST V2.12.0+ (ftp://ftp.ncbi.nlm.nih.gov/blast/executables/blast+/LATEST/) and InterProScan V5.56-89.0 of EMBL-EBI (https://www.ebi.ac.uk/interpro/search/sequence/).

### 4.4. Differentially Expressed Gene (DEG) Analysis

Clean reads were mapped to the reference sequences using HISAT2 software (version 2.1.0) with default parameters. The mapped reads were quantified using HTSeq (version 0.11.2, HTSeq documentation). Differential gene expression (DEG) analysis, the normalization of raw read counts, and log-fold change estimation were performed using DESeq2 (version 1.38.0, Bioconductor). Raw read counts were normalized using the SESeq2 normalization method implemented in the DESeq package in R software [37].

GO analysis using BLAST2GO V6.0.3 (https://www.blast2go.com/) was carried out using the sequence similarities (e-value ≤ 1 × 10^−10^) of proteins and classified into functional categories such as BP, CC, and MF. A heatmap was generated, showing the expression levels of HSF unigenes in the control and heat-shock-treated leave transcriptome using TBtools software (v2.080) [38]. Protein network analysis was performed to evaluate the degree of connectivity between the predicted amino-acid sequences obtained from a selected HSF TF sequence (*Gene_23847T*) and HSPs. The analysis was performed using STRING v11.0 (https://string-db.org/) with a minimum needed interaction score set at “medium confidence” (0.3).

### 4.5. cDNA Synthesis and Amplification of PgHSFA2

The *PgHSFA2* sequence was obtained from the ginseng transcriptome database available in the NCBI database. Total RNA was extracted from in vitro-cultured adventitious roots of ginseng using the RNeasy Plant Mini Kit (Qiagen). Complementary DNA (cDNA) was synthesized from the RNA using M-MLV Reverse Transcriptase (Invitrogen). The ORF of the *PgHSFA2* sequence was amplified from the cDNA by PCR. Primer sequences used for the amplification of *PgHSFA2* are provided in Table S1. The ORF region of the *PgHSFA2* gene was confirmed by resequencing the PCR products and has been registered in GenBank under accession number OQ134777.

### 4.6. Phylogenetic Analysis of the PgHSFA2 Gene

Phylogenetic analysis was conducted using the neighbor-joining method in MEGA 6.0 [39] with the deduced amino-acid sequences of *PgHSFA2* and homologous genes from other plants obtained from the DDBJ/GenBank/EMBL databases. The reliability of the tree nodes was evaluated through bootstrap analysis with 1000 replicates [40].

### 4.7. Abiotic Stress Treatments

To artificially induce heat stress, ginseng plants were placed in a 42 °C chamber for 0, 0.5, 1, 3, and 6 h, followed by a return to ambient temperature. For cold stress, ginseng plants were placed in a 4 °C chamber for the same time intervals (0, 0.5, 1, 3, and 6 h) and then returned to ambient conditions. Salt stress was simulated by transferring in vitro-cultured adventitious roots onto half-strength MS medium (without NH₄NO₃) supplemented with 3% sucrose, 0.3% Gelrite, and 200 mM NaCl, followed by incubation in a 24 °C chamber for 0, 0.5, 1, 3, 6, 12, and 24 h. A combined stress treatment of heat and sunlight was applied by exposing plants to outdoor conditions (approximately 32 °C) under full sunlight at midday for 3 h. After each treatment, the ginseng plants were immediately frozen in liquid nitrogen and stored at −70 °C.

### 4.8. RT-PCR and qRT-PCR for Analysis of the Relative Expression Level of PgHSFA2 Under Abiotic Stresses

mRNA was isolated from stressed ginseng leaves following the same protocol described previously. RT-PCR was performed using primers designed for the 969 bp partial sequence of *PgHSFA2*. The *PgActin* gene (500 bp partial sequence) was used as an internal control to confirm RNA integrity and loading accuracy.

Quantitative RT-PCR (qRT-PCR) was conducted using the Qiagen Rotor-Gene Q system (Qiagen, Hilden, Germany) with the Rotor-Gene SYBR Green PCR kit (Qiagen). The specificity of the reactions was confirmed by melting curve analysis. Relative mRNA levels for each sample were calculated using the 2^−ΔΔCt^ method and compared to those of unstressed plants. The qRT-PCR data were normalized against the *β-actin* gene of *P. ginseng* as an internal control. Primer sequences for *PgHSFA2* and *PgActin* used in qRT-PCR analyses are provided in Table S1. Each qRT-PCR analysis was performed in triplicate, and the data are presented as the mean of relative quantities ± standard error (SE).

### 4.9. Agrobacterium-Mediated Transformation for Generating PgHSFA2-Overexpressing Tobacco Plants

The *PgHSFA2* gene, driven by the CaMV 35S promoter, was cloned and inserted into the pB2GW7 vector containing the BASTA resistance gene (BAR) as a selection marker. Leaf explants from wild-type *Nicotiana tabacum* were inoculated with *Agrobacterium tumefaciens* GV3101 cells harboring the 35S:*PgHSFA2*-containing plasmid. The explants were cocultivated on half-strength MS medium supplemented with 3% sucrose, 0.3% Gelrite, and 2 mg L⁻¹ BA under dark conditions for three days. To eliminate bacteria, 300 mg L⁻¹ cefotaxime (Duchefa, Haarlem, The Netherlands) was added to the medium after cocultivation. Adventitious shoots emerging from the leaf margins were transferred to medium containing 25 mg L⁻¹ L-phosphinothricin (BASTA, Duchefa, Haarlem, The Netherlands) for the selection of putative transgenic plants.

Genomic DNA was extracted from the leaf tissue of surviving plants grown on BASTA-containing medium using the HiYield™ Genomic DNA Mini Kit (Plant) (RBC Bioscience, Seoul, Republic of Korea). Genomic-PCR analysis was performed using a DNA thermal cycler (Applied Biosystems) to confirm the insertion of the *PgHSFA2* gene into the tobacco genome. The primer sequences used for detecting *PgHSFA2* are provided in Table S1.

qRT-PCR analyses were conducted to evaluate the transgenic lines. The tobacco *β-actin* gene was used as an internal control to ensure RNA integrity and loading accuracy to normalize qRT-PCR data. Based on the qRT-PCR results, wild-type plants and three transgenic tobacco lines were transplanted into soil for further investigation. Primer sequences for the tobacco *β-actin* gene used in qRT-PCR are listed in Table S1.

### 4.10. Expression of NtHSPs in Wild-Type and Transgenic Tobacco Plants Under Normal and High-Temperature Conditions

In vitro-grown wild-type and transgenic plants were exposed to either ambient temperature (25 °C) or high temperature (42 °C) for 3 h in an incubation chamber. Collected leaves were immediately stored at −70 °C. Following RNA extraction and cDNA synthesis from both wild-type and transgenic lines, the relative expression levels of *NtHSP17.6*, *NtHSP18.2*, *NtHSP26*, *NtHSP70*, *NtHSP82*, *NtHSP90*, and *NtHSP101* were quantified by qPCR. Primer sequences for all *NtHSP* genes are provided in Table S1.

### 4.11. Resistance of Transgenic Tobacco Overexpressing PgHSFA2 Under Heat and Salt Stress Conditions

To evaluate the heat-stress resistance of tobacco plants, one-month-old transgenic lines overexpressing *PgHSFA2* (three independent lines) and wild-type plants grown in soil pots were subjected to 45 °C in a chamber for 3 h. After heat treatment, the plants were allowed to recover at 25 °C. Additionally, two-month-old transgenic tobacco plants (Tr-3 line) and wild-type plants were exposed to 50 °C in a chamber for 1 h, followed by recovery at 25 °C. Photographs of both transgenic and wild-type plants were taken 3 days after treatment.

For salt-tolerance assays, leaves from each transgenic line and wild-type plant were placed on half-strength MS solid medium containing 0, 200, or 400 mM NaCl. After 9 days, the leaves were photographed to assess the effects of salt stress.

### 4.12. Measurement of Chlorophyll Contents and Maximum Quantum Yield of PSII

The chlorophyll content of leaves from both transgenic and wild-type tobacco plants was measured under non-stressed conditions (0 mM NaCl or 25 °C) and stress conditions (200 or 400 mM NaCl for salt stress, or 42 °C for 3 and 6 h for heat stress) using a SPAD 502 Plus Chlorophyll Meter (Minolta SPAD-502, Osaka, Japan).

The photosynthetic efficiency of transgenic and wild-type plants was assessed by measuring the maximum quantum yield of PSII (Fv/Fm) using a FluorPen FP-100 (Somatco). Before measurement, leaves from each plant were adapted to the dark for 20 min. The experiment was conducted in triplicate and repeated three times.

### 4.13. Statistical Analysis

Statistical analysis was performed using SPSS software version 26 (SPSS Science, Chicago, IL, USA). Significant differences among means were determined using a one-way ANOVA, followed by Duncan’s post hoc test.

## 5. Conclusions

The transcriptional analysis of ginseng plants under heat shock revealed that *PgHSFA2* is one of the most responsive HSF genes. The *PgHSFA2* gene, identified as a class A HSF due to its two AHA motifs, shares similarities with *AtHsfA2* from *Arabidopsis*, a known key regulator of thermotolerance. *PgHSFA2* was strongly induced under heat, mirroring *AtHsfA2*’s stress-responsive behavior. The overexpression of *PgHSFA2* in transgenic tobacco enhanced heat and salinity tolerance by upregulating intrinsic tobacco HSPs under normal conditions. Transgenic tobacco maintained stable chlorophyll content and the maximum quantum efficiency of Photosystem II (PSII) under stress, indicating protection of the photosynthetic apparatus. These findings suggest that *PgHSFA2* plays a pivotal role in stress tolerance. Its overexpression in ginseng could improve varieties with enhanced resilience to abiotic stresses, offering valuable potential for agricultural applications.

## Data Availability

The transcriptome datasets generated in this study were deposited in the NCBI SRA (Sequence Read Archive) for the Bio project: https://www.ncbi.nlm.nih.gov/bioproject/?term=PRJNA1012566. (accessed on 4 September 2023).

## Supplementary Materials

The following supporting information can be downloaded at: https://www.mdpi.com/article/10.3390/ijms26083836/s1.
