# Supplementary material for: A Heat-Shock Transcription Factor in Panax ginseng, PgHSFA2, Confers Heat and Salt Resistance in Transgenic Tobacco
Source: Int J Mol Sci. 2025 Apr 18;26(8):3836. doi: 10.3390/ijms26083836 (PMC12028321; doi:10.3390/ijms26083836)
Supplement: Supplementary file 1 [file ijms-26-03836-s001.zip › Table S1.pdf]

Table S1. Primer sequences used in the present study

	TM (°C) / Length (bp)	Primer sequence
PgHSFA2 cloning-F	53.4 / 17	CCCTCCCCAAGCTTCTA
PgHSFA2 cloning-R	54.7 / 20	TCATGGATTCGACCTCAGAT
PgHSFA2 genomic-PCR-F	58.5 / 24	CAGAAAAGTGGATCCTGATCGATG
PgHSFA2 genomic-PCR-R	60.6 / 22	CAGCATAGCTTGTCCTCCTTGC
Bar genomic-PCR-F	59.4/20	ACATCGAGACAAGCACGGTC
Bar geniomic-PCR-R	58.8/18	TCCAGTCGTAGGCGTTGC
PgHSFA2-qPCR-F	59.6 / 21	ATGGTCAAGGCTGGATTTGCA
PgHSFA2-qPCR-R	58 / 22	GAGCCTCATCTCCAACATATGC
PgActin-qPCR-F	57.5 / 25	ACAAAAACAAAAACATTCCCTTCCT
PgActin-qPCR-R	57.4 / 21	ATTCATGCAGTCCCTCCATTG
NtHSP17.6-qPCR-F	55.6 / 20	CTGATTCCGAGCTTCTTTGG
NtHSP17.6-qPCR-R	55.8 / 20	CCAGGAAGATCCACCTTGAA
NtHSP18.2-qPCR-F	55.3 / 20	TCGAGTGCTCGAATTGATTG
NtHSP18.2-qPCR-R	56.9 / 20	TAAGGAACTTTCCGCTGCTC
NtHSP26-qPCR-F	57.3 / 21	GGCAGATGATGGACACTATGG
NtHSP26-qPCR-R	57.7 / 20	CCCAAGGAGCACGTATTTCC
NtHSP70-qPCR-F	56 / 22	ACTTAGAAGGTTGAGAACTGCT
NtHSP70-qPCR-R	55.4 / 20	CGGGTAATGGTGGAGTAGAA
NtHSP82-qPCR-F	55.7 / 22	GTCTTACATTGGATGCTGAACA
NtHSP82-qPCR-R	56.7 / 20	TGCTTACACAACAGGCTCAA
NtHSP90-qPCR-F	56.4 / 17	AGACTGCCCTCCTCACC
NtHSP90-qPCR-R	55.6 / 19	CCTCCTCCATCTTGCTACC
NtHSP101-qPCR-F	55.3 / 20	GCGATAGATTGCACCAAAGA
NtHSP101-qPCR-R	55.2 / 20	GCCCCAAGAAAAGGAATGAA
NtActin-qPCR-F	55.7 / 20	CTGCTGGAATTCACGAAACA
NtActin-qPCR-R	56.9 / 20	GCCACCACCTTGATCTTCAT
