# Supplementary material for: A Heat-Shock Transcription Factor in Panax ginseng, PgHSFA2, Confers Heat and Salt Resistance in Transgenic Tobacco
Source: Int J Mol Sci. 2025 Apr 18;26(8):3836. doi: 10.3390/ijms26083836 (PMC12028321; doi:10.3390/ijms26083836)
Supplement: Supplementary file 1 [file ijms-26-03836-s001.zip › Table S2.pdf]

Table S2. Selected top 15 unigenes showing highly increased read log2 fold chance after heat shock treatment among annotated unigenes in *P. ginseng* transcriptome

Unigene	DESeq Normalization value		log2 Fold Change	Description (accession number)
	Cont.	Heat shock		
Gene_22767	6.5	31185.4	12.2	22.0 kDa heat shock protein-like (XP_017228513.1)
Gene_02936	25.2	76502.5	11.6	heat shock 70 kDa protein (XP_011079384.1)
Gene_38422	0.8	2450.1	11.6	Unknown
Gene_33681	43.1	63030.6	10.5	heat shock 22 kDa protein, mitochondrial-like isoform X4 (XP_052208823.1)
Gene_30195	106.6	112965.2	10.0	17.3 kDa class II heat shock protein-like (XP_019237100.1)
Gene_13386	26.9	28289.2	10.0	pyridoxal 5'-phosphate synthase-like subunit PDX1.2 (XP_017231761.1)
Gene_37400	4.9	4272.3	9.8	Unknwon
Gene_29942	6.5	4843.7	9.5	protein BOBBER 1 (XP_027160555.1)
Gene_26728	116.4	85324.8	9.5	17.3 kDa class II heat shock protein (XP_009617035.1)
Gene_12403	0.8	591.0	9.5	putative nuclease HARBI1 (XP_017223139.1)
Gene_31859	27.7	14496.6	9.0	hypothetical protein (KAG6697511.1)
Gene_31940	0.8	426.4	9.0	protein heat-stress-associated 32 kDa (XP_050212847.1)
Gene_23541	2.4	1254.5	9.0	hypothetical protein (KDO55703.1)
Gene_37389	4.9	2289.1	8.9	uncharacterized protein (XP_028094161.1)
Gene_19932	29.3	12298.4	8.7	pentatricopeptide repeat-containing protein, chloroplastic (XP_019082003.1)
